# A comprehensive evaluation of collapsing methods using simulated and real data: excellent annotation of functionality and large sample sizes required

**DOI:** 10.3389/fgene.2014.00323

**Published:** 2014-09-15

**Authors:** Carmen Dering, Inke R. König, Laura B. Ramsey, Mary V. Relling, Wenjian Yang, Andreas Ziegler

**Affiliations:** ^1^Institut für Medizinische Biometrie und Statistik, Universität zu Lübeck, Universitätsklinikum Schleswig-HolsteinLübeck, Germany; ^2^Pharmaceutical Department, St. Jude Children's Research HospitalMemphis, TN, USA; ^3^Zentrum für Klinische Studien, Universität zu LübeckLübeck, Germany; ^4^School of Mathematics, Statistics and Computer Science, University of KwaZulu-NatalDurban, South Africa

**Keywords:** collapsing, rare variants, simulation study, comparison, burden test, *SLCO1B1*

## Abstract

The advent of next generation sequencing (NGS) technologies enabled the investigation of the rare variant-common disease hypothesis in unrelated individuals, even on the genome-wide level. Analysis of this hypothesis requires tailored statistical methods as single marker tests fail on rare variants. An entire class of statistical methods collapses rare variants from a genomic region of interest (ROI), thereby aggregating rare variants. In an extensive simulation study using data from the Genetic Analysis Workshop 17 we compared the performance of 15 collapsing methods by means of a variety of pre-defined ROIs regarding minor allele frequency thresholds and functionality. Findings of the simulation study were additionally confirmed by a real data set investigating the association between methotrexate clearance and the *SLCO1B1* gene in patients with acute lymphoblastic leukemia. Our analyses showed substantially inflated type I error levels for many of the proposed collapsing methods. Only four approaches yielded valid type I errors in all considered scenarios. None of the statistical tests was able to detect true associations over a substantial proportion of replicates in the simulated data. Detailed annotation of functionality of variants is crucial to detect true associations. These findings were confirmed in the analysis of the real data. Recent theoretical work showed that large power is achieved in gene-based analyses only if large sample sizes are available and a substantial proportion of causing rare variants is present in the gene-based analysis. Many of the investigated statistical approaches use permutation requiring high computational cost. There is a clear need for valid, powerful and fast to calculate test statistics for studies investigating rare variants.

## 1. Introduction

The common variant-common disease hypothesis has successfully been investigated using genome-wide association studies (GWAS) with unrelated individuals in the past decade (Saxena et al., 2007; Seng and Seng, 2008; Manolio, 2010; Speliotes et al., 2011). With the advent of high-throughput sequencing technologies, generally termed next generation sequencing (NGS) (Metzker, 2010), it has now become possible to efficiently study the rare variant-common disease hypothesis, even on the genome-wide level (Campbell and Manolio, 2007; Bodmer and Bonilla, 2008). Some variants may be very rare or even observed only once in the entire sample so that standard association analyzes are not reasonable. To make association analysis feasible, researchers have therefore aggregated rare variants from a genomic region of interest (ROI). In early successful studies, the number of variant sites in the ROI was counted and, subsequently, compared between affected and unaffected individuals (Fitze et al., 2002; Cohen et al., 2004).

These findings founded the basis for the development of sophisticated new statistical association methods, termed collapsing or burden methods, in which rare variants are not only counted but weighted in various ways. For example, variants may be weighted inversely proportional to their frequency (Madsen and Browning, 2009) because rare variants are more often predicted to be functional (Price et al., 2010). They have stronger effect sizes than common variants, which is consistent with the view that functional variants are subject to purifying selection pressure (Kryukov et al., 2007; Bodmer and Bonilla, 2008; Frazer et al., 2009). This idea has led to the development of four other branches of collapsing methods. In one, the putative functionality of the rare variant as predicted from software packages is included (Ng and Henikoff, 2001; Ramensky et al., 2002; Adzhubei et al., 2010). Another idea determines the weights of all variants empirically from their observed effect sizes (Zhang et al., 2011; Jiang et al., 2013). In the third approach, results from simulation studies of evolutionary models are utilized. The effect size, measured as odds ratio, is approximately inversely proportional to the square root of the minor allele frequency (MAF) (Price et al., 2010). Therefore, statistical power might be maximized using a flexible threshold for classifying variants as rare instead of a fixed threshold. Finally, an entirely different strategy was used by Luo et al. (2011). Following the model of Bickeböller et al. (Bickeböller and Thompson, 1996), they interpreted a chromosome as a continuum. This allows capturing the variation in an ROI of both rare and frequent variants.

To assess the value of a collapsing method, smaller scale simulation studies were usually reported in the articles proposing a new method. These studies demonstrated the potential high power of the burden methods. However, a comprehensive head-to-head comparison of many collapsing methods is still lacking. We close this gap by comparing 15 conceptually different collapsing methods (Table 1) using the simulation data set provided for the Genetic Analysis Workshop (GAW) 17 (Almasy et al., 2011). Findings from the analysis of real data set investigating methotrexate clearence in acute lymphoblastic leukemia (ALL) diseased children (Treviño et al., 2009) were in line with the conclusions drawn from the simulation study.

**Table 1:** **Properties of 15 collapsing methods: Year of publication, burden (B) or non-burden (NB) test, both common and rare variants used in the analysis [yes (Y)/no (N)], test considers different effect directions (Y/N), test able to handle the presence of non-causal variants (Y/N), phenotype quantitative (Q) and/or binary (B), covariates can be included (Y/N), *p*-values assessed by permutation (P) or from an asymptotic distribution (D)**.

**Method**	**Year**	**Burden/Non-burden**	**Common and rare**	**Direction of effect**	**Miss-classi-fication**	**Phenotype**	**Covariates**	**Permutation**
CAST	2007	B	N	N	N	B	N	D
CMC	2008	B	Y	N	N	B	N	P
RVT1	2009	B	N	N	N	B/Q	Y	D
RVT2	2009	B	N	N	N	B/Q	Y	D
WSS	2009	B	N	N	N	B	N	P
RC	2010	B	N	N	N	B	N	P
aSum	2010	NB	N	Y	N	B	N	P
VT	2010	B	N	Y	N	B/Q	Y	P
KBAC	2010	NB	N	N	Y	B	N	P
CMAT	2010	B	N	N	N	B	N	P
C-α	2011	NB	N	Y	Y	B	N	D
FPCA	2011	B	N	Y	N	B	Y	D
PWST	2011	NB	N	Y	N	B/Q	N	P
SKAT	2011	NB	Y	Y	Y	B/Q	Y	D
SKAT-O	2012	NB	Y	Y	Y	B/Q	Y	D

aSum, adaptive summation; CAST, cohort allelic sum test; CMAT, cumulative minor-allele test; CMC, combined multivariate cluster; FPCA, functional principal component analysis; KBAC, kernel-based adaptive cluster; PWST, p-value weighted sum test; RC, RARECOVER; RVT, rare variant test 1 and 2; SKAT, sequencing kernel association test; SKAT-O, optimal unified SKAT; VT, variable threshold; WSS, weighted sum statistic.

## 2. Materials and methods

### 2.1. Collapsing methods

All 15 methods require an ROI. An ROI may contain variants of an arbitrary genetic unit, e.g., a gene or a pathway. However, collapsing might be further refined in different ways. One approach to define an ROI is to use information on functionality or direction of the effect of a variant. These estimates may be obtained from software packages, such as Polyphen2 (Adzhubei et al., 2010), SIFT (Ng and Henikoff, 2001), SNPS3D (Yue et al., 2006), or PMUT (Ferrer-Costa et al., 2004). Most of the approaches intrinsically only consider variants with a MAF below a specific threshold, such as 1 or 5%, and only these variants form the ROI. However, since frequent variants are excluded by this definition, association information might be lost. Another group of approaches therefore investigates the joint effect of rare and common variants.

The early collapsing approaches all share the assumption that all variants below a certain MAF threshold are causal with the same direction of the effect, and these tests are termed burden tests. The simplest version of a burden test was used by Fitze et al. (2002) who counted the number of cases and the number of controls with at least one rare variant in an ROI. A slightly modified version, coined cohort allelic sum test (CAST), was proposed by Morgenthaler and Thilly (2007) who compared the number of minor alleles in an ROI between cases and controls. The cumulative minor-allele test (CMAT; Zawistowski et al., 2010) follows a similar idea but uses the odds ratio as measure of comparison, not the difference of counts. Li and Leal (2008) suggested to jointly investigate rare and common variants in an ROI; their approach is termed Combined Multivariate and Collapsing (CMC) test. Here, rare variants are pooled to one genetic unit, and the test is a multivariate test of both rare and common variants in an ROI. Two rare variant tests (RVT1, RVT2) followed similar ideas but used a generalized linear model (Morris and Zeggini, 2010). RVT2 uses the proportion of positions carrying at least one minor allele in an ROI as a weight of the expected increase in the phenotype, where RVT1 considers whether there is at least one position with a minor allele present in an ROI. Instead of using a fixed threshold for filtering by MAF, Price et al. (2010) introduced greater flexibility with the so-called Variable Threshold (VT) method which is the maximum of the test statistics over a range of MAFs. Bhatia et al. (2010) proposed a different maximum approach, named Rarecover (RC), and took the maximum of test statistics over a sliding window of chromosomal positions.

In the group of weighted sum statistics (WSS), variants are weighted inversely proportional to their MAF, e.g., in controls (Madsen and Browning, 2009), or using different weights involving cases and controls (Sul et al., 2011). An entirely different approach was suggested by Luo et al. (2011) who based their approach on the genome continuum model (Bickeböller and Thompson, 1996) and employed a principal components analysis (PCA) as basis for their Functional Principal Component Analysis (FPCA). In essence, the corresponding test statistic considers the mean-squared distance of averages of principal components scores between cases and controls.

More sophisticated tests, generally termed non-burden tests, are robust against presence of non-causal variants, generally named misclassification, and they may even deal with different effect directions of rare variants. For example, the issue of misclassification can be considered with the Kernel Based Adaptive Cluster (KBAC) method (Liu and Leal, 2010). Here, adaptive weights are introduced based on the sample risk of the structure of an ROI. Han and Pan (2010) proposed to consider different effect directions and suggested the use of data-adaptive weights, which were summed over all variants. Subsequently, their method is termed adaptive summation (aSum). Another method considering directional effects was introduced by Zhang et al. (2011), where weights for each variant are derived from a left-tail *p*-value of a single marker test. In C-α, directional effects are taken into account by using the difference between the observed and the expected variance of minor allele counts in cases (Neale et al., 2011). A method specialized to both the situation of the presence of non-causal, protective and deleterious variants is the Sequencing Kernel Association Test (SKAT) which uses a general regression framework together with variant-adaptive weights (Wu et al., 2011). Here, all individuals of the sample are compared pairwise for each variant with weights from the beta distribution. Since SKAT can be less powerful if the assumptions of a burden test are met, Lee et al. (2012) suggested the optimal unified test SKAT-O which combines burden and SKAT statistics.

### 2.2. Simulation data

Simulation data originate from the Genetic Analysis Workshop 17 (Almasy et al., 2011), where genotype data were taken from real data of the 1000 Genomes Project pilot3 study. Quality control of genotype data was conducted as part of the 1000 Genomes Project pilot3 study. Genotype data consist of 24, 487 exonic variants from 3205 genes in 697 unrelated individuals, originating from different populations. However, as described in Almasy et al. (2011), the phenotype was simulated independently of population origin, so that confounding by population is not present. Only 12.8% of the variants had MAF ≥ 0.05, and 74% of the variants had MAF ≤ 0.01; 9433 variants were private and occurred only once. Median MAF was 0.002.

Phenotype data were simulated for both binary and quantitative traits (Almasy et al., 2011). A common disease with prevalence of 30% and three related quantitative risk factors *Q*1, *Q*2, *Q*4 and smoking status were simulated. Quantitative risk factors were generated using the normal distribution. Disease status was simulated using a liability threshold model, and the top 30% of the distribution were declared affected so that there were 209 affected and 488 unaffected individuals in the sample. Phenotype simulations were repeated 200 times to generate 200 replicates; genotypes, age and sex were kept fix over replicates.

Associated genes were taken from the vascular endothelial growth factor (VEGF) pathway and from cardiovascular disease risk and inflammation causing genes. In total, 36 of the 3205 genes contained causal variants with a negative correlation between effect size and MAF.

Two criteria were used to collapse variants of a gene to one ROI. For functionality, only non-synonymous or gene-based variants were considered. For collapsing based on MAF threshold, variants were restricted to MAF thresholds of either 0.01 or 0.05. Furthermore, only chromosomal regions were considered, where variants were present at a minimum of two different positions. This resulted in four different collapsing scenarios which differed by the numbers of genes and variants (Table S1).

Most of the collapsing methods analyzed here test for association between an ROI and a binary affection status (Table 1). Only a few are designed to handle quantitative phenotypes or even covariates such as sex, age, or principal components to stratify for possible different population origins in the association test. If the method was designed to analyze a quantitative phenotype, we used *Q*2 as associated phenotype. If the method allowed for the inclusion of covariates, *Q*1, *Q*4, smoking status and age were utilized. However, in this simulation study some of the given method features were not considered due to software limitations or great computational costs. In case of RVT2, covariates were not included in the analysis because the software CCRaVaT does not allow for this feature. Furthermore, for VT and PWST, only the quantitative phenotype was considered because calculations for the affection status were computationally intractable. For SKAT and SKAT-O the small sample adjustment was used in the analysis.

### 2.3. *p*-value estimation of permutation tests

Many collapsing methods require permutation for obtaining *p*-values which are computer processing unit time intensive. We therefore used a four stage *p*-value estimation approach. Two thousand permutations were performed first, and an ROI was taken to the next higher number of permutations only if the estimated *p*-value was smaller than the upper bound of the corresponding confidence interval of the *p*-value. In the next stages, 10,000, 100,000, and 400,000 permutations were done to increase precision of *p*-value estimates.

### 2.4. Power and type I error

Within every replicate, we determined the proportion of not associated ROIs not exceeding the α-level. To estimate the type I error, this proportion was averaged over the 200 independent replicates. To evaluate whether an error was acceptable, we applied the threshold as suggested by Bradley (1978) of a liberal α-level of 1.5 × α with α = 0.05. Average (avg) power was calculated similarly via the average proportion of associated ROIs not exceeding the liberal α-level of 1.5 × 0.05 = 0.075. In addition, the minimal (min) power was computed as the average proportion of replicates where at least one associated ROI was detected.

### 2.5. Real data

In children with acute lymphoblastic leukemia (ALL), an association between methotrexate clearence and the *SLCO1B1* gene was detected using a GWAS (Treviño et al., 2009). Methotrexate is a drug used in the treatment of autoimmune diseases and malignancies inhibitating proliferating cells. In a follow up study, Ramsey et al. (2012) investigated the effect of rare variants on methotrexate clearence. They showed that the rare variants in the *SLCO1B1* gene had larger effect sizes than common variants.

To compare the 15 methods in this data set, genotype data of 673 children with ALL from different populations were made available. Phenotype data of methotraxate clearence were adjusted by sex, age, and genetic ancestry information. For the 5 collapsing methods investigating quantitative traits, information from all 673 individuals was used. For the methods analyzing binary traits, the first and the last decile of ordered adjusted methotrexate clearence were used to create two distinct groups (Risch and Zhang, 1995).

Collapsing was conducted with respect to variants with MAF < 0.05 and three ROIs of variants: all 93 gene variants, 15 non-synonymous variants and 7 of these non-synonymous variants, which were classified as damaging. Classification of damaging variants was conducted with the help of the prediction algorithms SIFT (Ng and Henikoff, 2001), PMUT (Ferrer-Costa et al., 2004), SNPS3D (Yue et al., 2006), and Polyphen2 (Adzhubei et al., 2010) as described in Ramsey et al. (2012) where variants were categorized as damaging if they were predicted to be damaging by at least three of the four algorithms. In case of permutation based tests 10^9^ permutations of phenotype data were conducted. Furthermore, a type I error-level of 0.05 was used.

## 3. Results

**Figure 1:**
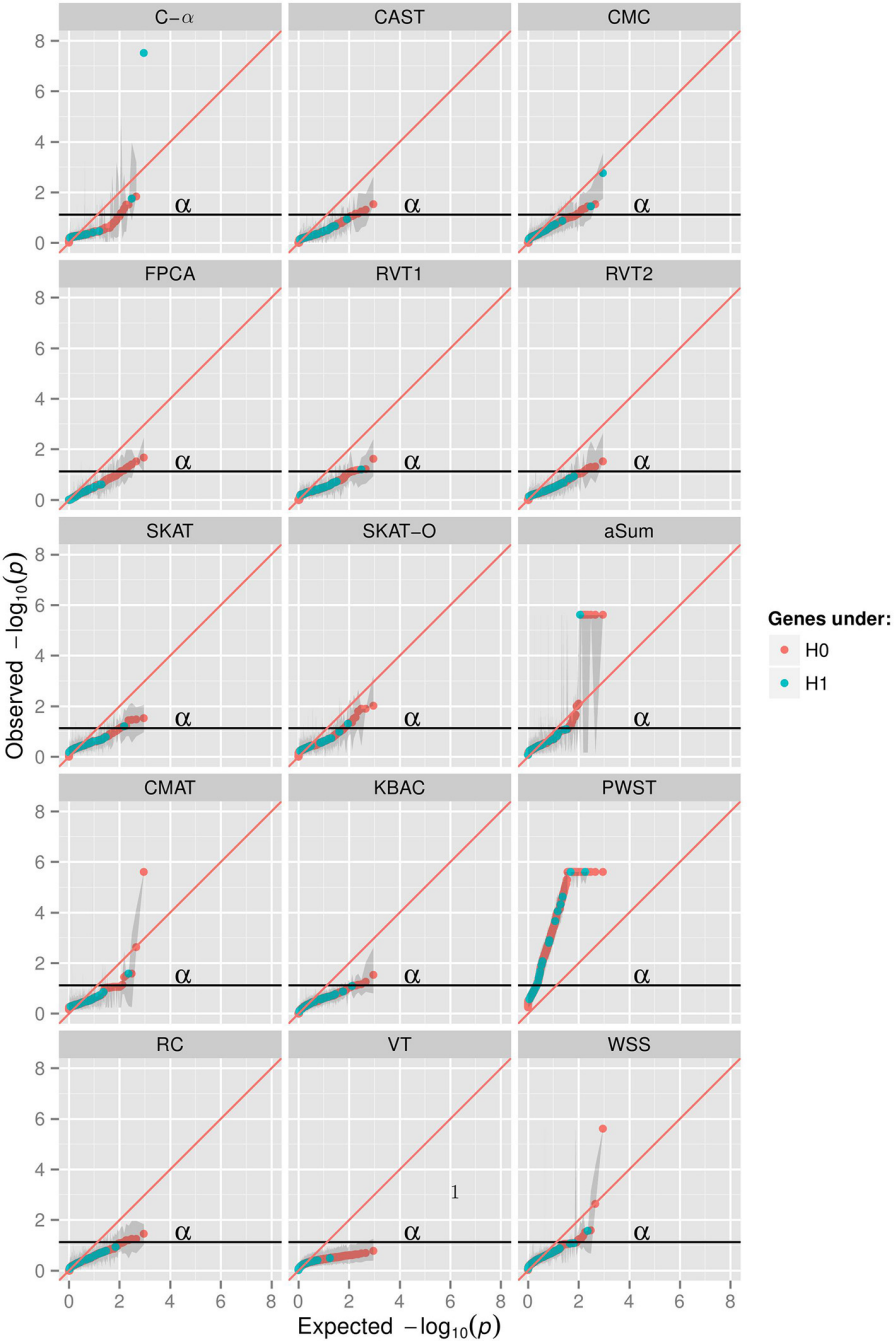
**Q-Q plots in 15 collapsing methods, minor allele frequency (MAF) threshold of 0.01, restriction to non-synonymuous variants, phenotype of affection status with no covariates; aSum, adaptive summation; CAST, cohort allelic sum test; CMAT, cumulative minor-allele test; CMC, combined multivariate cluster; FPCA, functional principal component analysis; KBAC, kernel-based adaptive cluster; PWST, *p*-value weighted sum test; RC, RARECOVER; RVT, rare variant test 1 and 2; SKAT, sequencing kernel association test; SKAT-O, optimal unified SKAT; VT, variable threshold; WSS, weighted sum statistic**. X-axis shows expected −10log transformed *p*-values from uniform distribution, y-axis shows observed median −10log transformed *p*-values of 200 replicates surrounded by a ribbon of the first and third quartile of *p*-values in 200 replicates.

The Q-Q plot (Figure 1) shows the median log transformed *p*-values surrounded by a gray ribbon indicating the first and third quartile of *p*-values which therefore does not reflect the entire range of *p*-values. It can be seen that none of the investigated collapsing methods on average revealed the expected uniform distribution of *p*-values under the null hypothesis of no association. Specifically, plotted observed vs. expected negative log *p*-values substantially deviated on average from the angle bisector. Furthermore, for many methods the type I error level was inflated, which is indicated by the gray ribbon surrounding the orange and blue dots in Figure 1. For illustration, Figure 1 displays the scenario with non-synonymous variants only, a MAF < 0.01 and the binary phenotype. However, findings for other scenarios were similar (Supplementary Material). The inflation of type I error levels can also be observed in Table 2, where findings are reported for the analysis of the affection status without adjustment for covariates, when the ROIs either included non-synonymous variants only or all gene-based variants and when collapsing was done with MAF < 0.01. In the scenario, when non-synonymous variants were considered, 5 tests had inflated type I error levels at the nominal 5% test-level. Of the methods with adequate type I error levels, CMAT had the highest min power of 1.00. The highest avg power of 0.13 was observed for C-α. When the ROI contained all variants of a gene with MAF < 0.01, more than 50% of the tests had inflated type I error levels. For the 7 tests with non-inflated type I error levels, power was higher compared to the previous scenario except of RVT2 for which power decreased. Results were similar when MAF threshold of 0.05 was used (Table S3). Results for the quantitative phenotype without covariates are provided in Table S4. Here, all collapsing methods except VT showed inflated type I error levels. When the model for the affection status included covariates, only RVT1 had adequate type I error levels with highest min power of 0.97 in the scenario of gene-based variants and MAF < 0.01 (Tables S5, S6). Here, aSum, PWST, CMC, SKAT, and SKAT-O revealed inflated type I error levels in all investigated scenarios, with PWST having type I error ≥ 0.50 (Table 2 and Supplementary Tables).

**Table 2:** **Type I error levels and power for collapsing with minor allele frequency < 0.01 for both non-synonymous and gene-based variants**.

**Variants**	**Method**	**Type I error**	**Avg. power**	**Min power**
Non-synonymous	aSum	0.12	(0.19)	(1.00)
	C-α	0.07	0.13	0.90
	CAST	0.06	0.11	0.93
	CMAT	0.05	0.10	1.00
	CMC	0.10	(0.16)	(0.93)
	FPCA	0.05	0.05	0.57
	KBAC	0.03	0.06	0.77
	PWST	0.50	(0.58)	(1.00)
	RC	0.07	0.12	0.93
	RVT1	0.05	0.11	0.77
	RVT2	0.06	0.12	0.93
	SKAT	0.08	(0.12)	(0.93)
	SKAT-O	0.10	(0.16)	(0.93)
	VT	0.07	0.04	0.70
	WSS	0.05	0.11	0.90
Gene-based	aSum	0.12	(0.15)	(1.00)
	C-α	0.06	0.10	0.91
	CAST	0.06	0.09	0.97
	CMAT	0.12	(0.16)	(1.00)
	CMC	0.11	(0.20)	(0.97)
	FPCA	0.05	0.07	0.82
	KBAC	0.04	0.06	0.88
	PWST	0.65	(0.85)	(1.00)
	RC	0.08	(0.13)	(0.94)
	RVT1	0.06	0.10	0.91
	RVT2	0.05	0.11	0.85
	SKAT	0.08	(0.11)	(0.97)
	SKAT-O	0.10	(0.14)	(1.00)
	VT	0.06	0.04	0.85
	WSS	0.12	(0.17)	(0.94)

Type I error levels and average (avg) power were averaged over 200 replicates. Minimal (min) power is the proportion of replicates for which at least one associated region of interest was detected. Power is given in parenthesis if the type I error was inflated.

aSum, adaptive summation; CAST, cohort allelic sum test; CMAT, cumulative minor-allele test; CMC, combined multivariate cluster; FPCA, functional principal component analysis; KBAC, kernel-based adaptive cluster; PWST, p-value weighted sum test; RC, RARECOVER; RVT, rare variant test 1 and 2; SKAT, sequencing kernel association test; SKAT-O, optimal unified SKAT; VT, variable threshold; WSS, weighted sum statistic.

Table 3 displays the results for the analyses of the real data set. For the ROI of damaging variants, all methods but CAST yielded *p*-values < 0.05. If the ROIs consisted in non-synonymous and gene-based variants, 6 and 4 tests had *p*-values < 0.05, respectively. Furthermore, except for PWST, FPCA, and SKAT, all methods had the lowest *p*-value when the ROI was defined using damaging variants only. For the majority of tests the highest *p*-value was obtained when ROIs were analyzed using non-synonymous variants only. In the subset of methods without inflated type I error levels (first four lines in Table 3), all methods had significant *p*-values in the ROI of damaging variants, and the lowest *p*-value was observed for C-α. When non-synonymous variants were considered, C-α and FPCA had *p*-values < 0.05 among the valid methods. When all variants from the gene were analyzed jointly, *p* < 0.05 only for C-α.

**Table 3:** ***p*-values from the association analysis between methotrexate clearence and the *SLCO1B1* gene in patients with acute lymphoblastic leukemia**.

**Method**	**Damaging**	**Non-synonymous**	**Gene-based**
C-α	1.13 · 10^−21^	1.63 · 10^−05^	9.93 · 10^−12^
FPCA	6.75 · 10^−06^	1.26 · 10^−06^	3.61 · 10^−01^
KBAC	2.89 · 10^−02^	4.46 · 10^−01^	3.44 · 10^−01^
VT	2.37 · 10^−04^	1.11 · 10^−01^	1.27 · 10^−01^
---------------------------------------------------------------			
CAST	5.79 · 10^−02^	7.91 · 10^−01^	6.02 · 10^−01^
RVT2	2.00 · 10^−03^	3.80 · 10^−01^	3.71 · 10^−01^
RVT1	1.30 · 10^−03^	7.79 · 10^−01^	5.36 · 10^−01^
CMAT	<1.0 · 10^−09^	8.74 · 10^−01^	6.69 · 10^−01^
RC	6.30 · 10^−07^	7.84 · 10^−06^	2.91 · 10^−06^
WSS	<1.0 · 10^−09^	6.13 · 10^−01^	1.97 · 10^−01^
aSum	5.30 · 10^−08^	1.14 · 10^−01^	1.02 · 10^−02^
CMC	1.76 · 10^−07^	3.77 · 10^−06^	1.03 · 10^−02^
PWST	1.36 · 10^−03^	1.18 · 10^−04^	1.95 · 10^−07^
SKAT	2.92 · 10^−02^	1.30 · 10^−02^	2.36 · 10^−01^
SKAT-O	2.38 · 10^−03^	5.77 · 10^−02^	4.04 · 10^−01^

Rare variants with minor allele frequency <0.05 were collapsed by functionality damaging, non-synonymous or gene-based. The number of permutations for permutation-based tests was 10^9^. Only the first four reported methods (separated by a dashed line) did not show inflated type I error levels in any of the scenarios from the simulation study and were therefore considered to be valid.

aSum, adaptive summation; CAST, cohort allelic sum test; CMAT, cumulative minor-allele test; CMC, combined multivariate cluster; FPCA, functional principal component analysis; KBAC, kernel-based adaptive cluster; PWST, p-value weighted sum test; RC, RARECOVER; RVT, rare variant test 1 and 2; SKAT, sequencing kernel association test; SKAT-O, optimal unified SKAT; VT, variable threshold; WSS, weighted sum statistic.

## 4. Discussion

In this comprehensive comparison of collapsing methods using simulated data from the GAW17 (Almasy et al., 2011), we unexpectedly found substantially inflated type I error levels for many of the proposed statistical methods. Specifically, only C-α, FPCA, KBAC, and VT had valid type I errors in all scenarios considered, which is low even given that there is a considerable chance to observe an inflated type I error in repeated measurements. In addition, the power to detect an association to a truly associated ROI was low for many of the methods when averaged over all 200 replicates. Over all investigated scenarios, avg power was not larger than 0.13, while min power ranged from 0.57 to 1.00. In the group of valid methods the largest min power and avg power was obtained by C-α in all four investigated scenarios with values of 0.90 and 0.13, respectively. It should be noted that besides the approach of a liberal α level for evaluating the type I error used in this work, further approaches have been suggested which may be applied to the values presented in Table 2 and the Supplementary Material. These might attenuate our conclusions regarding type I error at the cost of even decreased power. In the re-analysis of the real data on methotrexate clearance and variants in the *SLCO1B1* gene (Treviño et al., 2009; Ramsey et al., 2012), strong association signals were detected for many approaches, even for those methods without inflated type I errors levels in the simulation study. However, results varied substantially by the collapsing approach used. If analyses were restricted to damaging variants, *p*-values were generally lower than when non-synonymous variants were collapsed or when gene-based analyses were performed.

The low power of the association methods may be caused by the small sample size (209 cases, 488 controls) of the simulation study, and further studies are required to thoroughly investigate the effect of differing sample sizes on type I and II error. The simulation data possibly match the underlying assumptions of some methods better than those of others. Specifically, in the present simulation study, only positive effects of rare variants were simulated, thus fitting in with the assumptions of the burden tests. Therefore, results for burden tests might have been even more negative (and better for non-burden tests) if truly associated variants had been simulated with both positive and negative effects. In addition, disease status was simulated to be influenced by covariates such as smoking status and age. This aspect could only be considered by few of the considered methods in this work and may be a disadvantage for methods assuming only genetic effects. Despite these limitations, this work provides one of the most comprehensive performance comparisons of a wide range of collapsing concepts developed so far.

Most of the permutation-based tests were computationally very intensive. To give an example, the average calculation time of PWST for one ROI was about 20 min in one replicate, and this resulted in a CPU time of about 1900 days for the analysis of PWST in a single collapsing scenario.

An important aspect is that the inflated type I error levels have been reported for some collapsing methods before even by the authors of the original articles. Specifically, the *p*-value distribution of PWST, SKAT and SKAT-O observed here were in line with the results of the original work by Zhang et al. (2011) and Lee et al. (2012). These authors suggested adjustments to correct for the deviation of the *p*-value distribution from the angle bisector (Zhang et al., 2011; Lee et al., 2012) which did not yield a considerable improvement. Furthermore, both the simulation study and the analysis of the real data set confirm findings of Derkach et al. (2014) who stressed that the power of collapsing methods heavily relies on the proportion of causal variants in the considered ROI.

Our analyses showed that pre-information such as functionality or deleterious effects predicted from biotechnology software of the considered variants are extremely relevant to form ROIs for detecting true associations. Therefore, improvement and refinement of chosen ROIs for association analyses should be further developed. Moreover, performance of the considered collapsing methods in this work should be additionally investigated in studies with larger sample sizes.

Irrespective of the collapsing method used, studies with small to moderate sample sizes suffer from low power. Even in the case of sufficiently large samples, many of the investigated collapsing approaches might lack feasibility as they rely on permutations, requiring huge computational efforts. Large sample sizes, a substantial proportion of causing rare variants together with valid asymptotic methods are therefore required in gene-based analysis to reliably detect associations with large power.

## Author contributions

Carmen Dering and Andreas Ziegler designed the study. Carmen Dering performed the analyses. Results were critically reviewed by all authors. Carmen Dering and Andreas Ziegler wrote the first draft of the manuscript, which was critically reviewed by all other authors. Laura B. Ramsey, Mary V. Relling and Wenjian Yang collected the real data set and conducted the whole corresponding pre-analysis and annotation. All authors have seen and approved this final version of the manuscript.

### Conflict of interest statement

Andreas Ziegler is member of the editorial board of the Biometrical Journal, Human Genetics, Methods of Information in Medicine and Statistics in Medicine. The authors declare that the research was conducted in the absence of any commercial or financial relationships that could be construed as a potential conflict of interest.

## References

[B1] Adzhubei I. A. Schmidt S. Peshkin L. Ramensky V. E. Gerasimova A. Bork P. (2010). A method and server for predicting damaging missense mutations. Nat. Methods 7, 248–249 10.1038/nmeth0410-248 20354512 PMC2855889

[B2] Almasy L. Dyer T. D. Peralta J. M. Kent J. W. Jr. Charlesworth J. C. Curran J. E. (2011). Genetic analysis workshop 17 mini-exome simulation. BMC Proc. 5(Suppl. 9):S2 10.1186/1753-6561-5-S9-S2 22373155 PMC3287854

[B3] Bhatia G. Bansal V. Harismendy O. Schork N. J. Topol E. J. Frazer K. (2010). A covering method for detecting genetic associations between rare variants and common phenotypes. PLoS Comput. Biol. 6:e1000954 10.1371/journal.pcbi.1000954 20976246 PMC2954823

[B4] Bickeböller H. Thompson E. A. (1996). The probability distribution of the amount of an individual's genome surviving to the following generation. Genetics 143, 1043–1049 8725249 10.1093/genetics/143.2.1043 PMC1207322

[B5] Bodmer W. Bonilla C. (2008). Common and rare variants in multifactorial susceptibility to common diseases. Nat. Genet. 40, 695–701 10.1038/ng.f.136 18509313 PMC2527050

[B6] Bradley J. V. (1978). Robustness? Br. J. Math. Stat. Psychol. 31, 144–152 10.1111/j.2044-8317.1978.tb00581.x

[B7] Campbell H. Manolio T. (2007). Commentary: rare alleles, modest genetic effects and the need for collaboration. Int. J. Epidemiol. 36, 445–448 10.1093/ije/dym055 17470492

[B8] Cohen J. C. Kiss R. S. Pertsemlidis A. Marcel Y. L. McPherson R. Hobbs H. H. (2004). Multiple rare alleles contribute to low plasma levels of HDL cholesterol. Science 305, 869–872 10.1126/science.1099870 15297675

[B9] Derkach A. Lawless J. F. Sun L. (2014). Pooled association tests for rare genetic variants: a review and some new results. Stat. Sci. 29, 302–321 10.1214/13-STS456

[B10] Ferrer-Costa C. Orozco M. de la Cruz X. (2004). Sequence-based prediction of pathological mutations. Proteins 57, 811–819 10.1002/prot.20252 15390262

[B11] Fitze G. Cramer J. Ziegler A. Schierz M. Schreiber M. Kuhlisch E. (2002). Association between c135g/a genotype and RET proto-oncogene germline mutations and phenotype of hirschsprung's disease. Lancet 359, 1200–1205 10.1016/S0140-6736(02)08218-1 11955539

[B12] Frazer K. A. Murray S. S. Schork N. J. Topol E. J. (2009). Human genetic variation and its contribution to complex traits. Nat. Rev. Genet. 10, 241–251 10.1038/nrg2554 19293820

[B13] Han F. Pan W. (2010). A data-adaptive sum test for disease association with multiple common or rare variants. Hum. Hered. 70, 42–54 10.1159/000288704 20413981 PMC2912645

[B14] Jiang Y. Epstein M. P. Conneely K. N. (2013). Assessing the impact of population stratification on association studies of rare variation. Hum. Hered. 76, 28–35 10.1159/000353270 23921847 PMC4406348

[B15] Kryukov G. V. Pennacchio L. A. Sunyaev S. R. (2007). Most rare missense alleles are deleterious in humans: implications for complex disease and association studies. Am. J. Hum. Genet. 80, 727–739 10.1086/513473 17357078 PMC1852724

[B16] Lee S. Emond M. J. Bamshad M. J. Barnes K. C. Rieder M. J. Nickerson D. A. (2012). Optimal unified approach for rare-variant association testing with application to small-sample case-control whole-exome sequencing studies. Am. J. Hum. Genet. 91, 224–237 10.1016/j.ajhg.2012.06.007 22863193 PMC3415556

[B17] Li B. Leal S. (2008). Methods for detecting associations with rare variants for common diseases: application to analysis of sequence data. Am. J. Hum. Genet. 83, 311–321 10.1016/j.ajhg.2008.06.024 18691683 PMC2842185

[B18] Liu D. J. Leal S. M. (2010). A novel adaptive method for the analysis of next-generation sequencing data to detect complex trait associations with rare variants due to gene main effects and interactions. PLoS Genet. 6:e1001156 10.1371/journal.pgen.1001156 20976247 PMC2954824

[B19] Luo L. Boerwinkle E. Xiong M. (2011). Association studies for next-generation sequencing. Genome Res. 21, 1099–1108 10.1101/gr.115998.110 21521787 PMC3129252

[B20] Madsen B. E. Browning S. R. (2009). A groupwise association test for rare mutations using a weighted sum statistic. PLoS Genet. 5:e1000384 10.1371/journal.pgen.1000384 19214210 PMC2633048

[B21] Manolio T. A. (2010). Genomewide association studies and assessment of the risk of disease. N.Engl. J. Med. 363, 166–176 10.1056/NEJMra0905980 20647212

[B22] Metzker M. L. (2010). Sequencing technologies - the next generation. Nat. Rev. Genet. 11, 31–46 10.1038/nrg2626 19997069

[B23] Morgenthaler S. Thilly W. G. (2007). A strategy to discover genes that carry multi-allelic or mono-allelic risk for common diseases: a cohort allelic sums test (CAST). Mutat. Res. 615, 28–56 10.1016/j.mrfmmm.2006.09.003 17101154

[B24] Morris A. P. Zeggini E. (2010). An evaluation of statistical approaches to rare variant analysis in genetic association studies. Genet. Epidemiol. 34, 188–193 10.1002/gepi.20450 19810025 PMC2962811

[B25] Neale B. M. Rivas M. A. Voight B. F. Altshuler D. Devlin B. Orho-Melander M. (2011). Testing for an unusual distribution of rare variants. PLoS Genet. 7:e1001322 10.1371/journal.pgen.1001322 21408211 PMC3048375

[B26] Ng P. C. Henikoff S. (2001). Predicting deleterious amino acid substitutions. Genome Res. 11, 863–874 10.1101/gr.176601 11337480 PMC311071

[B27] Price A. L. Kryukov G. V. de Bakker P. I. W. Purcell S. M. Staples J. Wei L.-J. (2010). Pooled association tests for rare variants in exon-resequencing studies. Am. J. Hum. Genet. 86, 832–838 10.1016/j.ajhg.2010.04.005 20471002 PMC3032073

[B28] Ramensky V. Bork P. Sunyaev S. (2002). Human non-synonymous SNPs: server and survey. Nucleic Acids Res. 30, 3894–3900 10.1093/nar/gkf493 12202775 PMC137415

[B29] Ramsey L. B. Bruun G. H. Yang W. Treviño L. R. Vattathil S. Scheet P. (2012). Rare versus common variants in pharmacogenetics: SLCO1b1 variation and methotrexate disposition. Genome Res. 22, 1–8 10.1101/gr.129668.111 22147369 PMC3246196

[B30] Risch N. Zhang H. (1995). Extreme discordant sib pairs for mapping quantitative trait loci in humans. Science 268, 1584–1589 10.1126/science.7777857 7777857

[B31] Saxena R. Voight B. F. Lyssenko V. Burtt N. P. Bakker P. I. W. D. Chen H. (2007). Genome-wide association analysis identifies loci for type 2 diabetes and triglyceride levels. Science 316, 1331–1336 10.1126/science.1142358 17463246

[B32] Seng K. C. Seng C. K. (2008). The success of the genome-wide association approach: a brief story of a long struggle. Eur. J. Hum. Genet. 16, 554–564 10.1038/ejhg.2008.12 18285837

[B33] Speliotes E. K. Yerges-Armstrong L. M. Wu J. Hernaez R. Kim L. J. Palmer C. D. (2011). Genome-wide association analysis identifies variants associated with nonalcoholic fatty liver disease that have distinct effects on metabolic traits. PLoS Genet. 7:e1001324 10.1371/journal.pgen.1001324 21423719 PMC3053321

[B34] Sul J. H. Han B. He D. Eskin E. (2011). An optimal weighted aggregated association test for identification of rare variants involved in common diseases. Genetics 188, 181–188 10.1534/genetics.110.125070 21368279 PMC3120154

[B35] Treviño L. R. Shimasaki N. Yang W. Panetta J. C. Cheng C. Pei D. (2009). Germline genetic variation in an organic anion transporter polypeptide associated with methotrexate pharmacokinetics and clinical effects. J. Clin. Oncol. 27, 5972–5978 10.1200/JCO.2008.20.4156 19901119 PMC2793040

[B36] Wu M. C. Lee S. Cai T. Li Y. Boehnke M. Lin X. (2011). Rare-variant association testing for sequencing data with the sequence kernel association test. Am. J. Hum. Genet. 89, 82–93 10.1016/j.ajhg.2011.05.029 21737059 PMC3135811

[B37] Yue P. Melamud E. Moult J. (2006). SNPs3d: candidate gene and SNP selection for association studies. BMC Bioinformatics 7:166 10.1186/1471-2105-7-166 16551372 PMC1435944

[B38] Zawistowski M. Gopalakrishnan S. Ding J. Li Y. Grimm S. Zllner S. (2010). Extending rare-variant testing strategies: analysis of noncoding sequence and imputed genotypes. Am. J. Hum. Genet. 87, 604–617 10.1016/j.ajhg.2010.10.012 21070896 PMC2978957

[B39] Zhang Q. Irvin M. R. Arnett D. K. Province M. A. Borecki I. (2011). A data-driven method for identifying rare variants with heterogeneous trait effects. Genet. Epidemiol. 35, 679–685 10.1002/gepi.20618 21818776 PMC3201701

