# Supplementary material for: A comprehensive evaluation of collapsing methods using simulated and real data: excellent annotation of functionality and large sample sizes required
Source: Front Genet. 2014 Sep 15;5:323. doi: 10.3389/fgene.2014.00323 (PMC4164031; doi:10.3389/fgene.2014.00323)
Supplement: Supplementary file 1 [file DataSheet1.PDF]

Frontiers in Statistical Genetics and Methodology

Research Article
13 August 2014

Supplementary Material: A comprehensive evaluation of collapsing methods using simulated and real data: Excellent annotation of functionality and large sample sizes required

Carmen Dering [1], Inke R König [1] Laura B Ramsey [2], Mary V Relling [2], Wenjian Yang [2] and Andreas Ziegler [1,3,*]

[1] *Institut für Medizinische Biometrie und Statistik, Universität zu Lübeck, Universitätsklinikum Schleswig-Holstein, Campus Lübeck, Lübeck, Germany*
[2] *Pharmaceutical Department St. Jude Children's Research Hospital, Memphis, TN USA*
[3] *Zentrum für Klinische Studien, Universität zu Lübeck, Germany*

Correspondence*:
Andreas Ziegler
Institut für Medizinische Biometrie und Statistik, Universität zu Lübeck, Universitätsklinikum Schleswig-Holstein, Campus Lübeck, Ratzeburger Allee 160, 23562 Lübeck, Germany, ziegler@imbs.uni-luebeck.de

Identification of rare genetic variants contributing to human diseases

1 SUPPLEMENTARY TABLES AND FIGURES

TABLES

Table S1. Scenario summaries of simulation data on investigated region of interests (ROI) with respect to minor allele frequency (MAF), number of genes and number of variants.

MAF	ROI	# genes	# variants
0.01	non-synonymous	909	7085
0.05	non-synonymous	1014	8182
0.01	gene-based	1140	11677
0.05	gene-based	1328	13747

Table S2. Utilized software in the analysis of 15 collapsing methods.

Test	Software
aSum	AssotesteR R-package
CMAT	AssotesteR R-package
KBAC	KBAC R-package
PWST	own R-implementation
RC	rvtests github project
VT	rvtests github project
WSS	own R-implementation
CAST	AssotesteR R-package
C-α	AssotesteR R-package
CMC	AssotesteR R-package
FPCA	Luo R-Code
RVT1	GRANVIL
RVT2	CCRaVaT/QuiTie
SKAT	SKAT R-package
SKAT-O	SKAT R-package

aSum: adaptive summation; CAST: cohort allelic sum test; CMAT: cumulative minor-allele test; CMC: combined multivariate cluster; FCPA: functional principal component analysis; KBAC: kernel-based adaptive cluster; PWST: p-value weighted sum test; RC: RARECOVER; RVT: rare variant test 1 and 2; SKAT: sequencing kernel association test; SKAT-O: optimal unified SKAT; VT: variable threshold; WSS: weighted sum statistic.

Table S3. Power values and type I error in 15 collapsing methods: Collapsing with respect to minor allele frequency < 0.01 and non-synonymous and gene-based variants; type I error and average (avg) Power values are averaged over 200 replicates, minimal (min) Power is defined as the proportion of replicates for which at least one associated region of interest was detected; Power values in parentheses due to inflated type I error.

Variants	Method	Type-I-Error	avg Power	min Power
	aSum	0.12	(0.19)	(1.00)
	C-alpha	0.07	0.13	0.90
	CAST	0.06	0.11	0.93
	CMAT	0.05	0.10	1.00
	CMC	0.10	(0.16)	(0.93)
	FPCA	0.05	0.05	0.57
	KBAC	0.03	0.06	0.77
non-synonymous	PWST	0.50	(0.58)	(1.00)
	RC	0.07	0.12	0.93
	RVT1	0.05	0.11	0.77
	RVT2	0.06	0.12	0.93
	SKAT	0.08	(0.12)	(0.93)
	SKAT-O	0.10	(0.16)	(0.93)
	VT	0.07	0.04	0.70
	WSS	0.05	0.11	0.90
	aSum	0.12	(0.15)	(1.00)
	C-alpha	0.06	0.10	0.91
	CAST	0.06	0.09	0.97
	CMAT	0.12	(0.16)	(1.00)
	CMC	0.11	(0.20)	(0.97)
	FPCA	0.05	0.07	0.82
	KBAC	0.04	0.06	0.88
gene-based	PWST	0.65	(0.85)	(1.00)
	RC	0.08	(0.13)	(0.94)
	RVT1	0.06	0.10	0.91
	RVT2	0.05	0.11	0.85
	SKAT	0.08	(0.11)	(0.97)
	SKAT-O	0.10	(0.14)	(1.00)
	VT	0.06	0.04	0.85
	WSS	0.12	(0.17)	(0.94)

aSum: adaptive summation; CAST: cohort allelic sum test; CMAT: cumulative minor-allele test; CMC: combined multivariate cluster; FCPA: functional principal component analysis; KBAC: kernel-based adaptive cluster; PWST: p-value weighted sum test; RC: RARECOVER; RVT: rare variant test 1 and 2; SKAT: sequencing kernel association test; SKAT-O: optimal unified SKAT; VT: variable threshold; WSS: weighted sum statistic.

Table S4. Power values and type I error with respect to quantitative phenotype of in 6 collapsing methods: p-value weighted sum test (PWST), rare variant tests 1 and 2 (RVT1, RVT2) and sequencing kernel association test (SKAT), the optimal unified SKAT (SKAT-O) and variable threshold (VT); type I error and avg Power values are averaged over 200 replicates, min Power is defined as the proportion of replicates for which at least one associated region of interest was detected; Power values in parentheses due to inflated type I error.

MAF	Variants	Method	Type-I-Error	avg Power	min Power
0.01	non-synonymous	PWST	0.50	(0.58)	(1.00)
		RVT1	0.09	(0.20)	(1.00)
		RVT2	0.09	(0.21)	(1.00)
		SKAT	0.09	(0.18)	(1.00)
		SKAT-O	0.10	(0.21)	(1.00)
		VT	0.07	0.04	0.70
	gene-based	PWST	0.65	(0.85)	(1.00)
		RVT1	0.10	(0.17)	(1.00)
		RVT2	0.10	(0.16)	(1.00)
		SKAT	0.10	(0.18)	(1.00)
		SKAT-O	0.10	(0.19)	(1.00)
		VT	0.06	0.04	0.85
0.05	non-synonymous	PWST	0.58	(0.68)	(1.00)
		RVT1	0.10	(0.19)	(1.00)
		RVT2	0.10	(0.20)	(1.00)
		SKAT	0.09	(0.18)	(1.00)
		SKAT-O	0.10	(0.21)	(1.00)
		VT	0.07	0.04	0.71
	gene-based	PWST	0.61	(0.84)	(1.00)
		RVT1	0.11	(0.17)	(1.00)
		RVT2	0.11	(0.17)	(1.00)
		SKAT	0.10	(0.18)	(1.00)
		SKAT-O	0.11	(0.19)	(1.00)
		VT	0.06	0.03	0.76

RVT: rare variant test 1 and 2; SKAT: sequencing kernel association test; SKAT-O: optimal unified SKAT; VT: variable threshold.

Table S5. Power values and type I error with respect to quantitative phenotype with covariates of in 3 collapsing methods: Type I error and avg Power values are averaged over 200 replicates, min Power is defined as the proportion of replicates for which at least one associated region of interest was detected; Power values in due to inflated type I error.

MAF	Variants	Method	Type-I-Error	avg Power	min Power
0.01	non-synonymous	RVT1	0.09	(0.20)	(1.00)
		SKAT	0.09	(0.18)	(1.00)
		SKAT-O	0.10	(0.21)	(1.00)
	gene-based	RVT1	0.10	(0.17)	(1.00)
		SKAT	0.10	(0.17)	(1.00)
		SKAT-O	0.10	(0.19)	(1.00)
0.05	non-synonymous	RVT1	0.10	(0.19)	(1.00)
		SKAT	0.09	(0.18)	(1.00)
		SKAT-O	0.10	(0.21)	(1.00)
	gene-based	RVT1	0.11	(0.17)	(1.00)
		SKAT	0.10	(0.17)	(1.00)
		SKAT-O	0.11	(0.18)	(1.00)

RVT1: rare variant test 1; SKAT: sequencing kernel association test; SKAT-O: optimal unified SKAT.

Table S6. Power values and type I error with respect to phenotype of affection status with covariates of in 3 collapsing methods: Type I error and avg Power values are averaged over 200 replicates, min Power is defined as the proportion of replicates for which at least one associated region of interest was detected; Power values in parentheses due to inflated type I error.

MAF	Variants	Method	Type-I-Error	avg Power	min Power
0.01	non-synonymous	RVT1	0.05	0.10	0.80
		SKAT	0.08	(0.12)	(1.00)
		SKAT-O	0.10	(0.17)	(0.97)
	gene-based	RVT1	0.06	0.11	0.97
		SKAT	0.08	(0.11)	(1.00)
		SKAT-O	0.10	(0.15)	(1.00)
0.05	non-synonymous	RVT1	0.07	0.16	0.87
		SKAT	0.09	(0.17)	(1.00)
		SKAT-O	0.11	(0.20)	(0.97)
	gene-based	RVT1	0.08	(0.17)	(0.97)
		SKAT	0.10	(0.16)	(1.00)
		SKAT-O	0.11	(0.19)	(1.00)

RVT1: rare variant test 1; SKAT: sequencing kernel association test; SKAT-O: optimal unified SKAT.

1.1 FIGURES

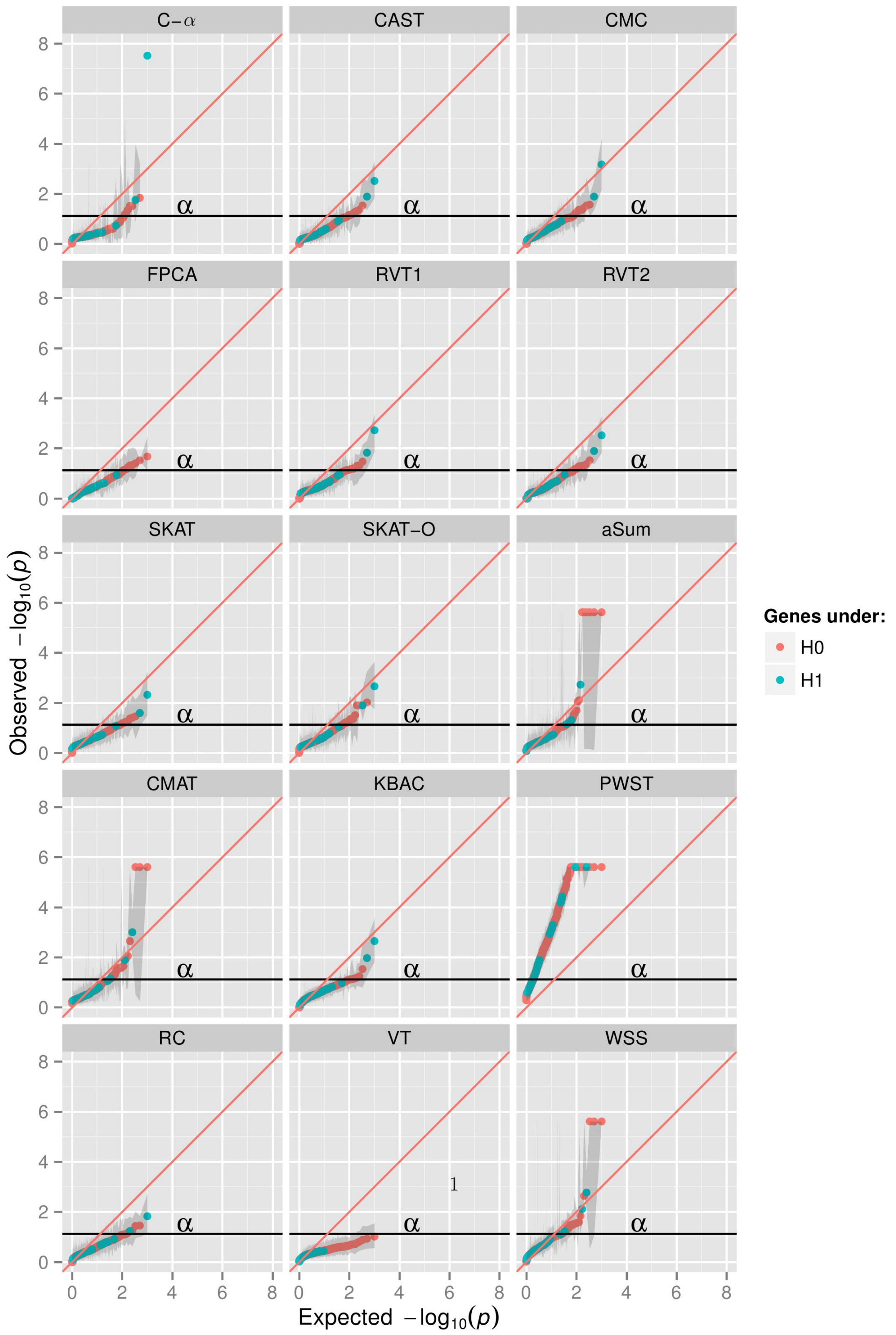

Figure S1. Q-Q plots in 15 collapsing methods, minor allele frequency (MAF) threshold of 0.05, restriction to non-synonymuous variants: aSum: adaptive summation; CAST: cohort allelic sum test; CMAT: cumulative minor-allele test; CMC: combined multivariate cluster; FCPA: functional principal component analysis; KBAC: kernel-based adaptive cluster; PWST: p-value weighted sum test; RC: RARECOVER; RVT: rare variant test 1 and 2; SKAT: sequencing kernel association test; SKAT-O: optimal unified SKAT; VT: variable threshold; WSS: weighted sum statistic. X-axis shows expected $-10\log$ transformed p-values from uniform distribution, y-axis shows observed median $-10\log$ transformed p-values of 200 replicates surrounded by a ribbon of the first and third quartile of p-values in 200 replicates.

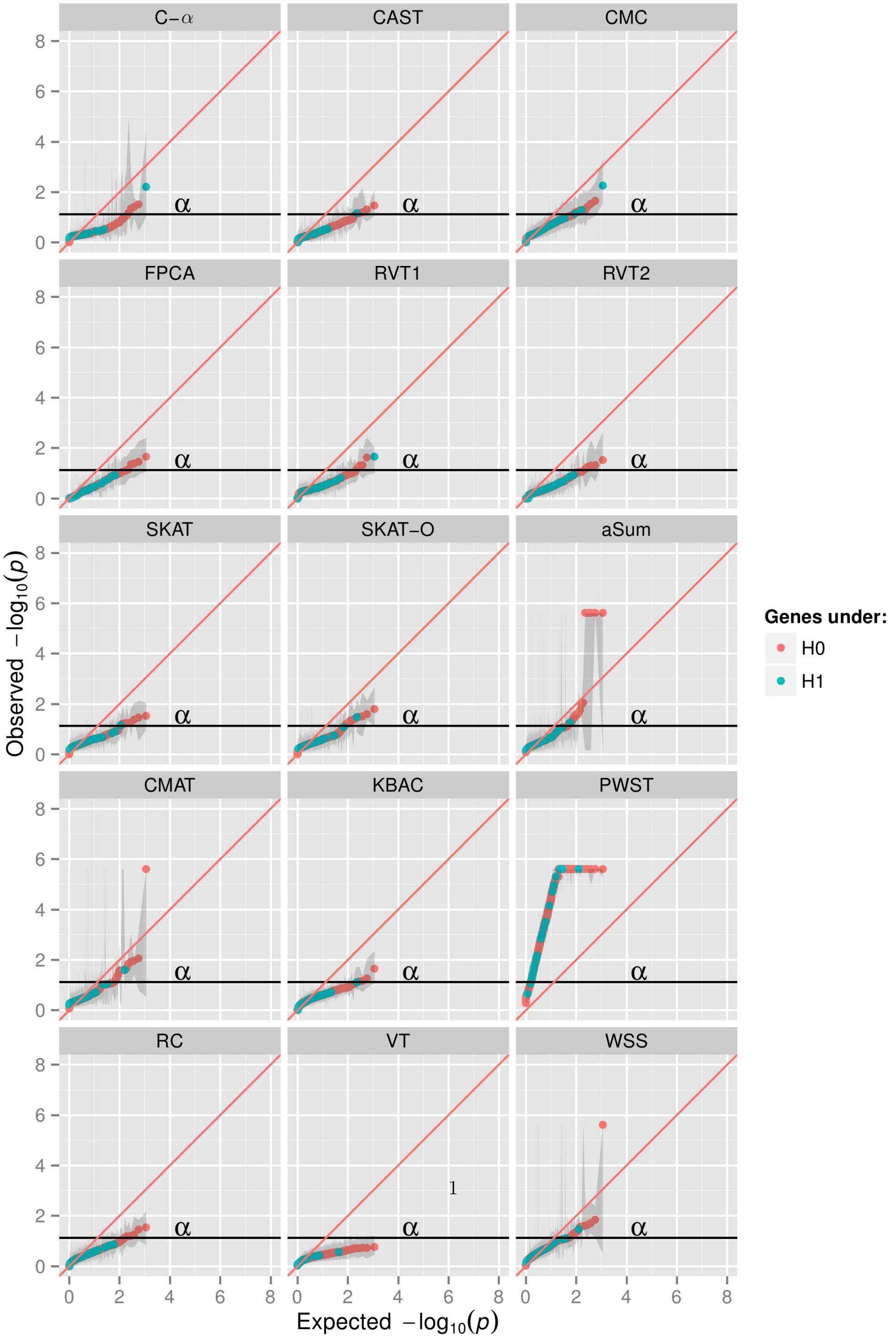

Figure S2. Q-Q plots in 15 collapsing methods, minor allele frequency (MAF) threshold of 0.01, all gene variants: aSum: adaptive summation; CAST: cohort allelic sum test; CMAT: cumulative minor-allele test; CMC: combined multivariate cluster; FCPA: functional principal component analysis; KBAC: kernel-based adaptive cluster; PWST: p-value weighted sum test; RC: RARECOVER; RVT: rare variant test 1 and 2; SKAT: sequencing kernel association test; SKAT-O: optimal unified SKAT; VT: variable threshold; WSS: weighted sum statistic. X-axis shows expected −10log transformed p-values from uniform distribution, y-axis shows observed median −10log transformed p-values of 200 replicates surrounded by a ribbon of the first and third quartile of p-values in 200 replicates.

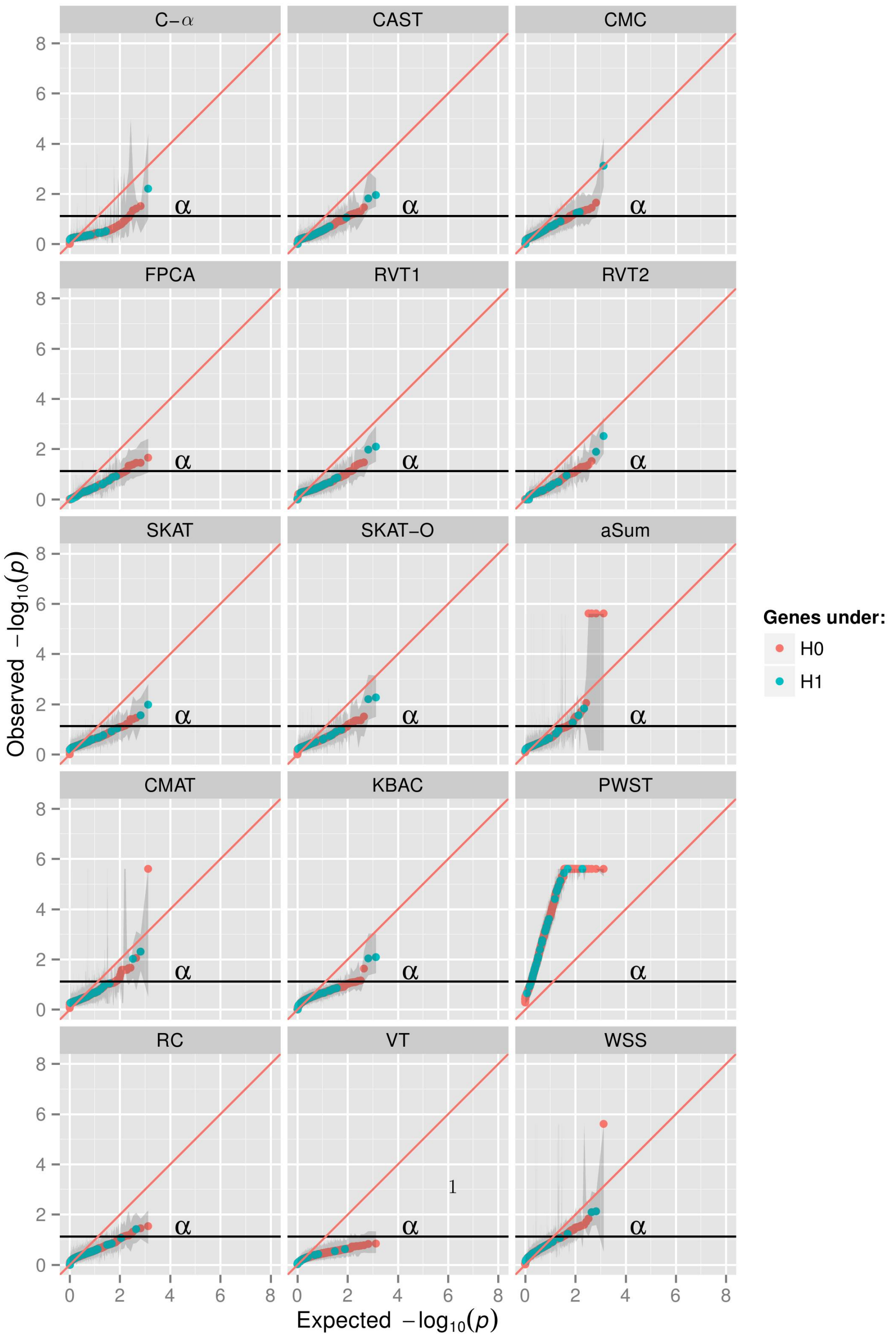

Figure S3. Q-Q plots in 15 collapsing methods, minor allele frequency (MAF) threshold of 0.05, no restriction to gene variants: aSum: adaptive summation; CAST: cohort allelic sum test; CMAT: cumulative minor-allele test; CMC: combined multivariate cluster; FCPA: functional principal component analysis; KBAC: kernel-based adaptive cluster; PWST: p-value weighted sum test; RC: RARECOVER; RVT: rare variant test 1 and 2; SKAT: sequencing kernel association test; SKAT-O: optimal unified SKAT; VT: variable threshold; WSS: weighted sum statistic. X-axis shows expected $-10\log$ transformed p-values from uniform distribution, y-axis shows observed median $-10\log$ transformed p-values of 200 replicates surrounded by a ribbon of the first and third quartile of p-values in 200 replicates.

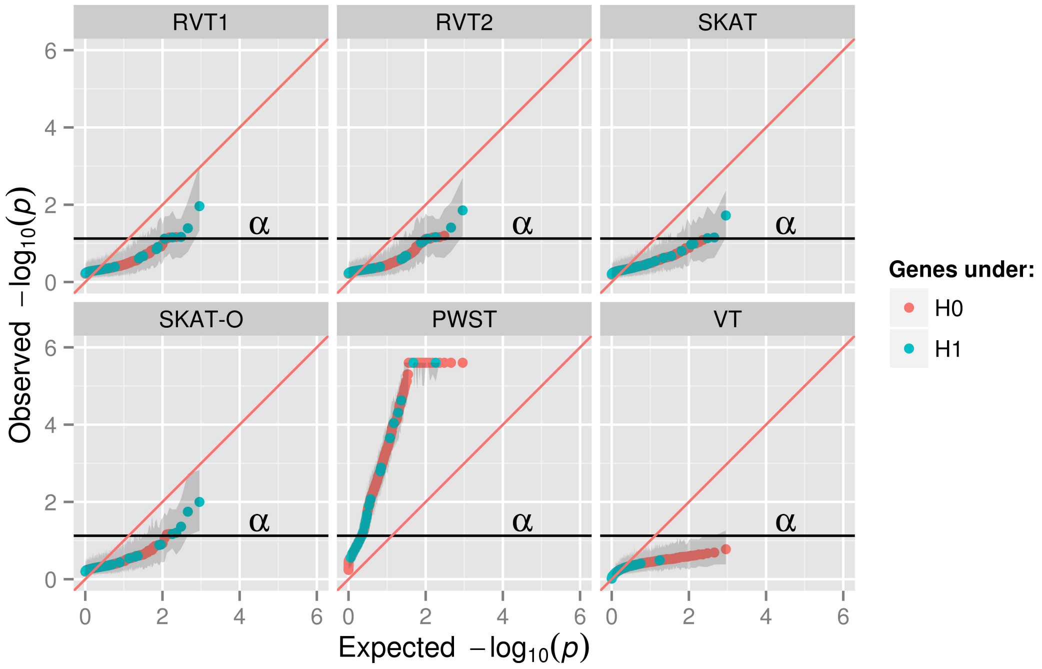

Figure S4. Q-Q plots in in 6 collapsing methods, minor allele frequency (MAF) threshold of 0.01, restriction to non-synonymuous variants, quantitative phenotype with no covariates; RVT: rare variant test 1 and 2; SKAT: sequencing kernel association test; SKAT-O: optimal unified SKAT; VT: variable threshold. X-axis shows expected -10log transformed p-values from uniform distribution, y-axis shows observed median -10log transformed p-values of 200 replicates surrounded by a ribbon of the first and third quartile of p-values in 200 replicates.

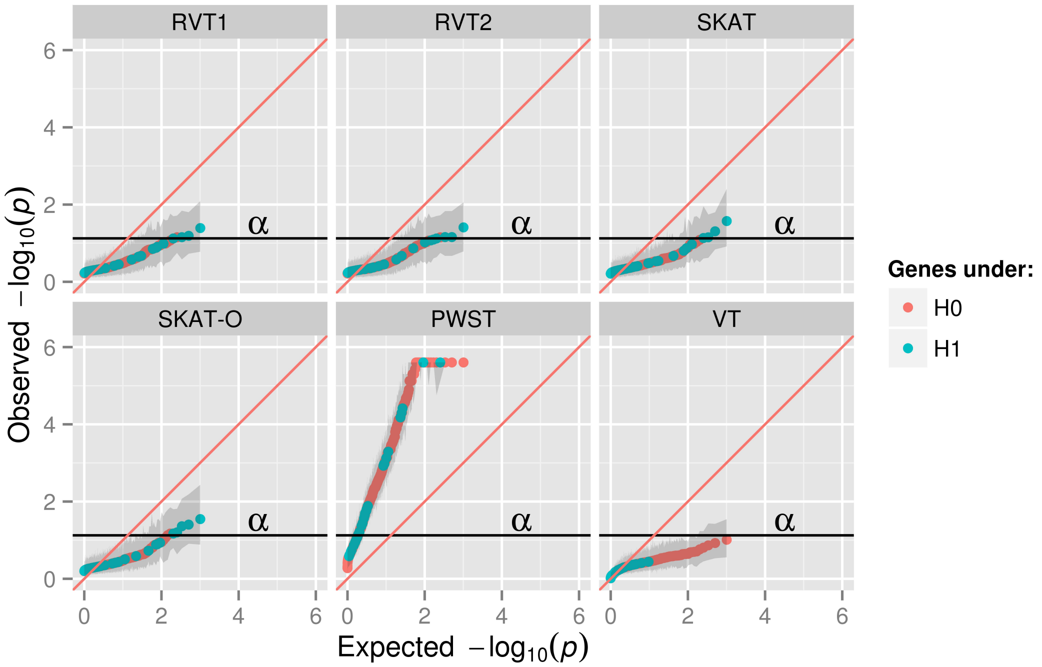

Figure S5. Q-Q plots in 6 collapsing methods, minor allele frequency (MAF) threshold of 0.05, with restriction to non-synonymous variants and with respect to the quantitative phenotype with no covariates: RVT: rare variant test 1 and 2; SKAT: sequencing kernel association test; SKAT-O: optimal unified SKAT; VT: variable threshold. X-axis shows expected -10log transformed p-values from uniform distribution, y-axis shows observed median -10log transformed p-values of 200 replicates surrounded by a ribbon of the first and third quartile of p-values in 200 replicates.

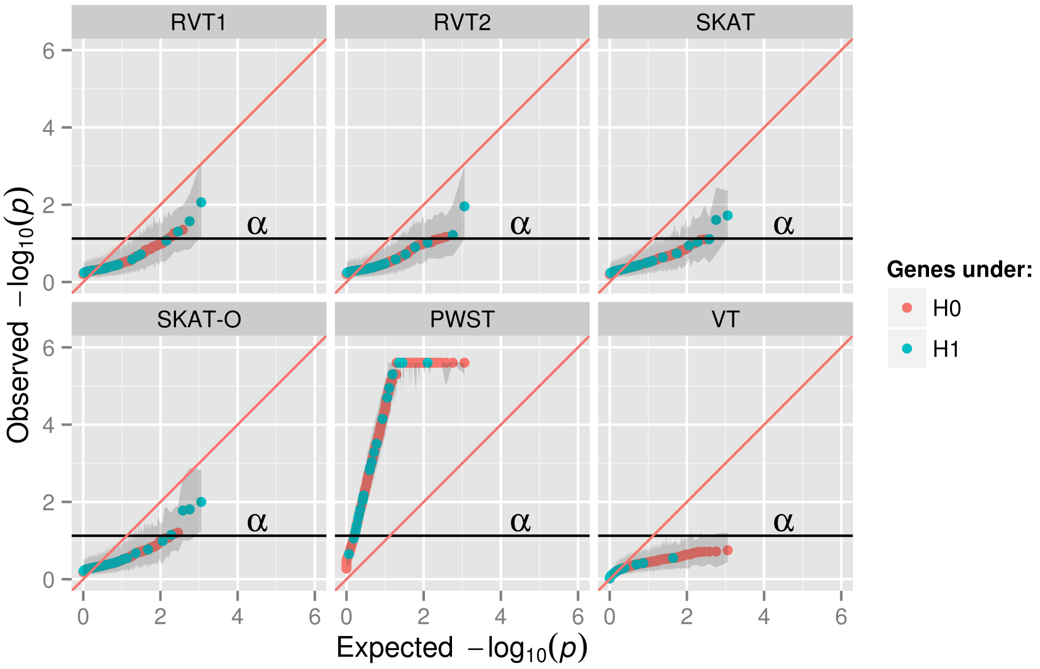

Figure S6. Q-Q plots in 6 collapsing methods, minor allele frequency (MAF) threshold of 0.01, no restriction to gene-based variants and with respect to the quantitative phenotype with no covariates: RVT: rare variant test 1 and 2; SKAT: sequencing kernel association test; SKAT-O: optimal unified SKAT; VT: variable threshold. X-axis shows expected -10log transformed p-values from uniform distribution, y-axis shows observed median -10log transformed p-values of 200 replicates surrounded by a ribbon of the first and third quartile of p-values in 200 replicates.

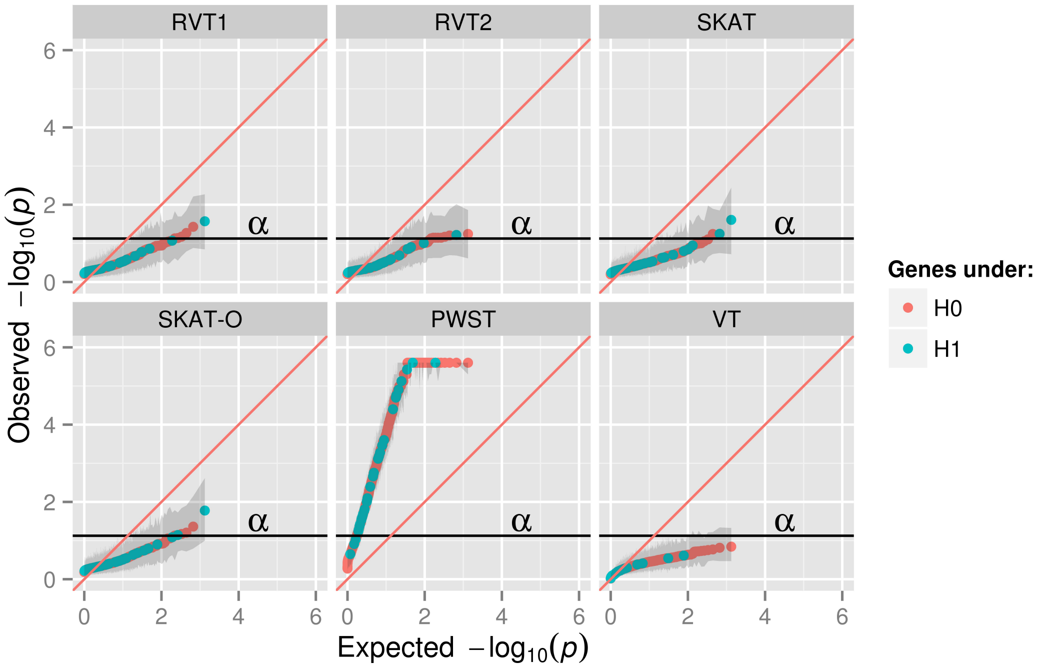

Figure S7. Q-Q plots in 6 collapsing methods minor allele frequency (MAF) threshold of 0.05, no restriction to gene-based variants and with respect to the quantitative phenotype with no covariates: RVT: rare variant test 1 and 2; SKAT: sequencing kernel association test; SKAT-O: optimal unified SKAT; VT: variable threshold. X-axis shows expected -10log transformed p-values from uniform distribution, y-axis shows observed median -10log transformed p-values of 200 replicates surrounded by a ribbon of the first and third quartile of p-values in 200 replicates.

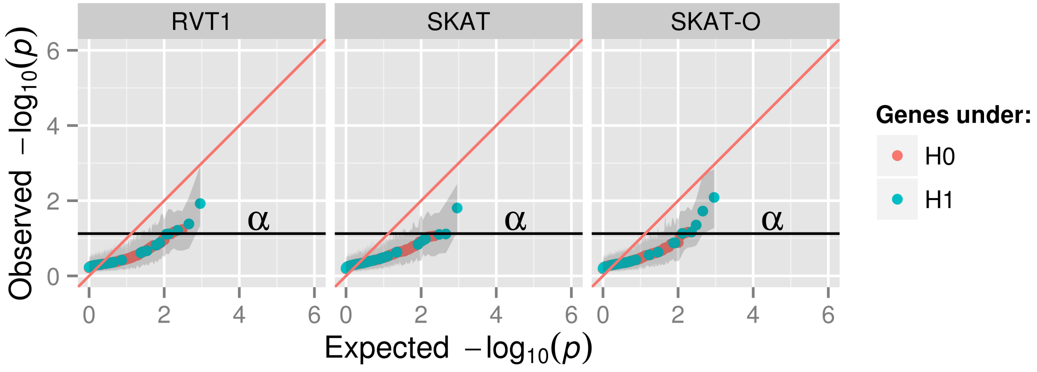

Figure S8. Q-Q plots in in 3 collapsing methods, minor allele frequency (MAF) threshold of 0.01, restriction to non-synonymuous variants, quantitative phenotype with covariates: RVT1: rare variant test 1; SKAT: sequencing kernel association test; SKAT-O: optimal unified SKAT. X-axis shows expected -10log transformed p-values from uniform distribution, y-axis shows observed median -10log transformed p-values of 200 replicates surrounded by a ribbon of the first and third quartile of p-values in 200 replicates.

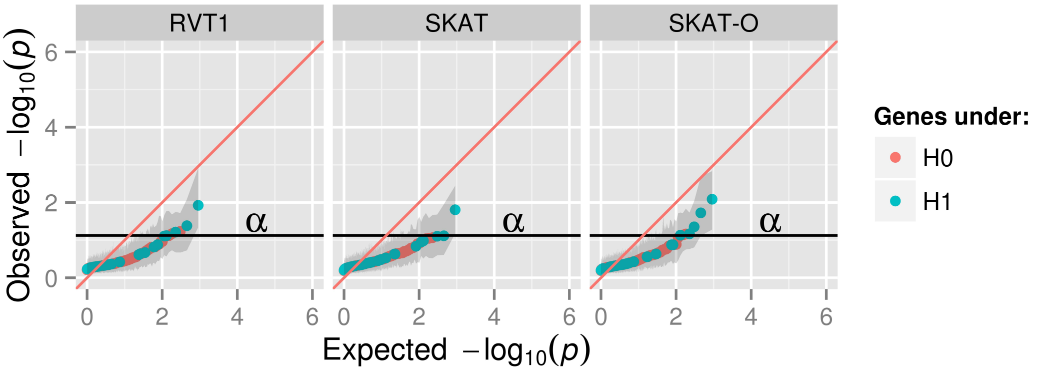

Figure S9. Q-Q plots in 3 collapsing methods, minor allele frequency (MAF) threshold of 0.05, with restriction to non-synonymous variants and with respect to the quantitative phenotype with covariates: RVT1: rare variant test 1; SKAT: sequencing kernel association test; SKAT-O: optimal unified SKAT. X-axis shows expected −10log transformed p-values from uniform distribution, y-axis shows observed median −10log transformed p-values of 200 replicates surrounded by a ribbon of the first and third quartile of p-values in 200 replicates..

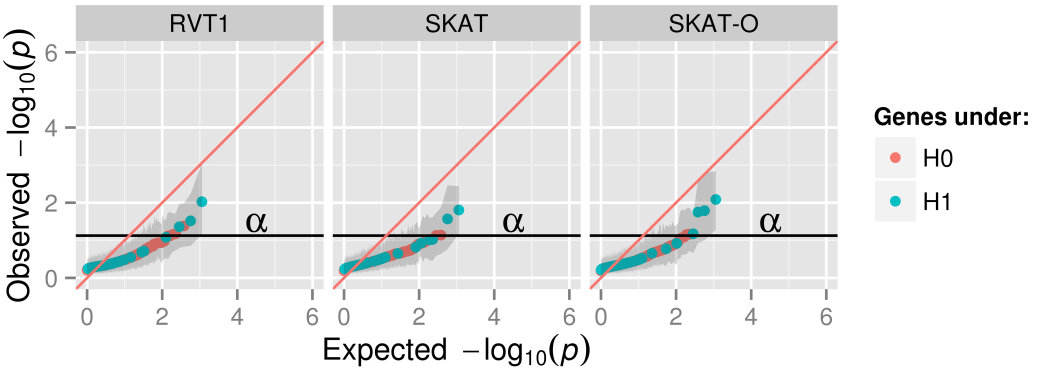

Figure S10. Q-Q plots in 3 collapsing methods, minor allele frequency (MAF) threshold of 0.01, no restriction to gene-based variants and with respect to the quantitative phenotype with covariates: RVT1: rare variant test 1; SKAT: sequencing kernel association test; SKAT-O: optimal unified SKAT. X-axis shows expected −10log transformed p-values from uniform distribution, y-axis shows observed median −10log transformed p-values of 200 replicates surrounded by a ribbon of the first and third quartile of p-values in 200 replicates.

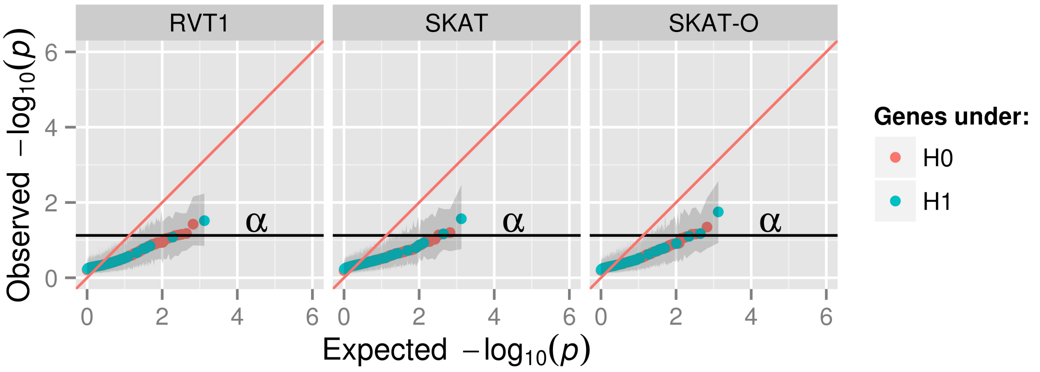

Figure S11.Q-Q plots in 3 collapsing methods, minor allele frequency (MAF) threshold of 0.05, no restriction to gene-based variantsand with respect to the quantitative phenotype with covariates: RVT1: rare variant test 1; SKAT: sequencing kernel association test; SKAT-O: optimal unified SKAT. X-axis shows expected -10log transformed p-values from uniform distribution, y-axis shows observed median -10log transformed p-values of 200 replicates surrounded by a ribbon of the first and third quartile of p-values in 200 replicates.

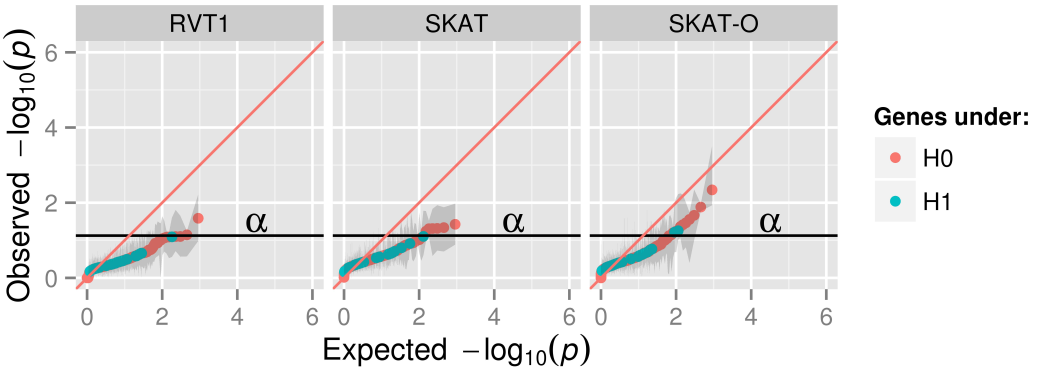

Figure S12. Q-Q plots in in 3 collapsing methods, minor allele frequency (MAF) threshold of 0.01, restriction to non-synonymuous variants, binary phenotype with covariates: RVT1: rare variant test 1; SKAT: sequencing kernel association test; SKAT-O: optimal unified SKAT. X-axis shows expected -10log transformed p-values from uniform distribution, y-axis shows observed median -10log transformed p-values of 200 replicates surrounded by a ribbon of the first and third quartile of p-values in 200 replicates.

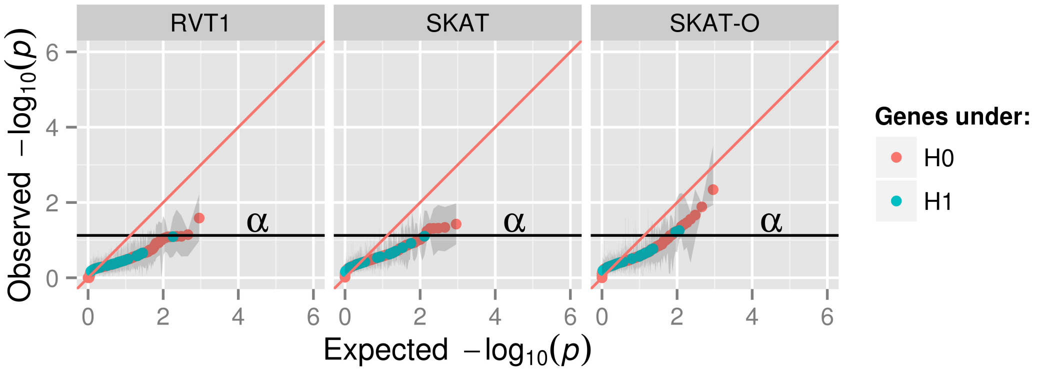

Figure S13. Q-Q plots in 3 collapsing methods, minor allele frequency (MAF) threshold of 0.05, with restriction to non-synonymous variants and with respect to the phenotype of affection status with covariates: RVT1: rare variant test 1; SKAT: sequencing kernel association test; SKAT-O: optimal unified SKAT. X-axis shows expected $-10\log$ transformed p-values from uniform distribution, y-axis shows observed median $-10\log$ transformed p-values of 200 replicates surrounded by a ribbon of the first and third quartile of p-values in 200 replicates.

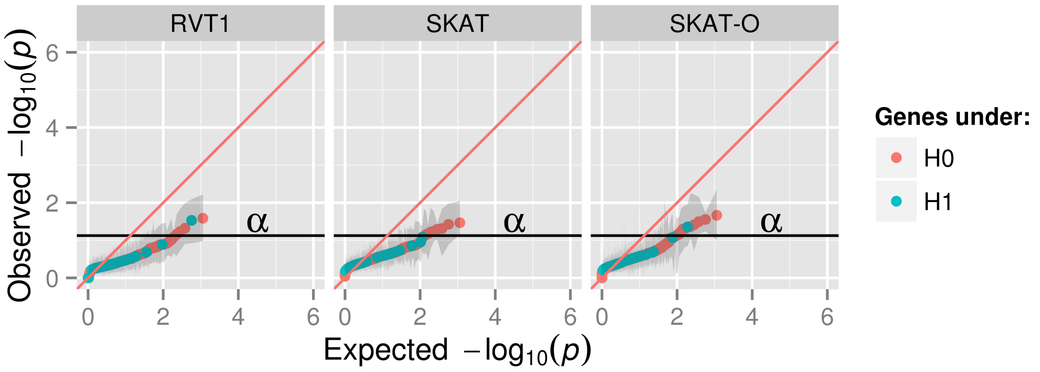

Figure S14. Q-Q plots in 3 collapsing methods, minor allele frequency (MAF) threshold of 0.01, no restriction to gene-based variants and with respect to the the phenotype of affection status with covariates: RVT1: rare variant test 1; SKAT: sequencing kernel association test; SKAT-O: optimal unified SKAT. X-axis shows expected $-10\log$ transformed p-values from uniform distribution, y-axis shows observed median $-10\log$ transformed p-values of 200 replicates surrounded by a ribbon of the first and third quartile of p-values in 200 replicates.

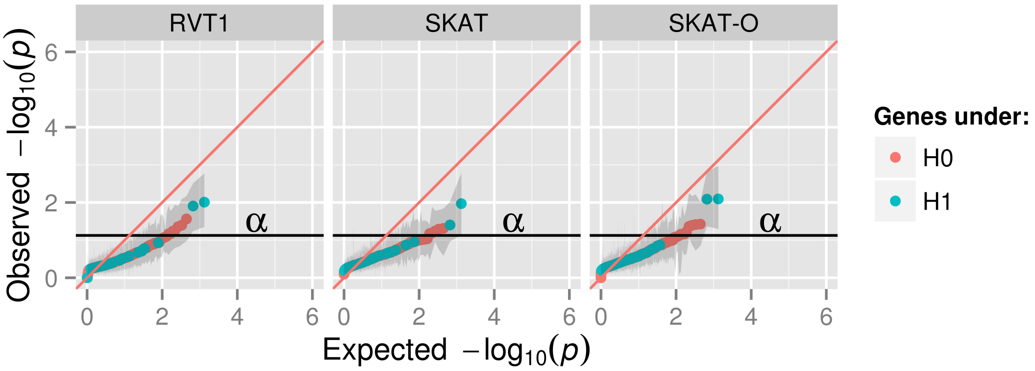

Figure S15. Q-Q plots in 3 ycollapsing methods, minor allele frequency (MAF) threshold of 0.05, no restriction to gene-based variants and with respect to the the phenotype of affection status with covariates: RVT1: rare variant test 1; SKAT: sequencing kernel association test; SKAT-O: optimal unified SKAT. X-axis shows expected $-10\log$ transformed p-values from uniform distribution, y-axis shows observed median $-10\log$ transformed p-values of 200 replicates surrounded by a ribbon of the first and third quartile of p-values in 200 replicates.
